# Confidence Region Identification and Contour Detection in MRI Image

**DOI:** 10.1155/2022/5898479

**Published:** 2022-08-08

**Authors:** Khurram Ejaz, Muhammad Arif, Mohd Shafry Mohd Rahim, Diana Izdrui, Daniela Maria Craciun, Oana Geman

**Affiliations:** ^1^Department of Computer Science and Information Technology, University of Lahore, Lahore, Pakistan; ^2^Department of Computer Science, University Technology Malaysia, Skudai, Malaysia; ^3^Electrical Engineering and Computer Science Faculty, Stefan Cel Mare University Suceava, Suceava, Romania; ^4^Faculty of Medicine and Biomedical Sciences, Stefan Cel Mare University Suceava, Suceava, Romania

## Abstract

Tumour region extraction (RE) method identifies the area of interest in MR imaging as it also highlights tumour boundaries. Some other intensities are existing, they are not visible but have their existence in region, and this region is called growing region. Such region is to be tumour region. Due to the variation of intensities in MRI images, tumour visibility becomes uncleared. Tumour intensity variations (tumour tissues) mix with normal brain tissues. In the light of above circumstance, tumour growing region becomes challenge. The goal of work is to extract the region of interest with confidence. The objective of the study is to develop the region of interest of brain tumour MRI image method by using confidence score for identifying the variation of intensity. The significance of work is based on identification of region of interest (tumour region). Confidence score is measured through pattern of intensities of MRI image. Similar patterns of brain tumour intensities are identified. Each pattern of intensities is adjusted with certain scale, and then biggest blob is analysed. Various biggest area blobs are combined, and resultant biggest blob is formed. In fact, resultant area blob is a combination of different patterns. Each pattern is assigned with particular colour. These colours highlight the growing region. Further, a contour is detected around the tumour boundaries. With combination of region scale fitting and contour detection (CD), tumour boundaries are further separated from normal tissues. Hence, the confidence score (CS) is formed from CD. CS is further converted to confidence region (CR). Conversion to CR is performed though confidence interval (CI). CI is based on defined conditions. In such conditions, different probabilities are considered. Probability identifies the region. Source of region formation is pixels; these pixels highlight tumour core significantly. This CR is obtained through checking standard deviation and statistical evaluation using confidence interval. Hence, region-of-interest pixels are identifying the CR. CR is evaluated through 97% Dice over index (DOI), 94% Jacquard, MSE 1.24, and PSNR 17.45. Value of testing parameter from benchmark study was JI, DOI, and MSE, PSNR : JI was 31.5%, DOI was 47.3%, MSE was 2.5 dB, and PSNR was 40 dB. The parameters are measured for the complex images; contribution parameter classifies the mean pixel values and deviating pixel values, and the classification of the pixel value is like to be termed as intensities. Mentioned classification extracts the variation of intensity pixels accurately; then, algorithm is highlighting the region as compared to the normal tumour cells.

## 1. Introduction

The region of interest (ROI) gives the border of an object under certain circumstances; certain processes are required to be performed over an object. The object pertains a tumour region, where the tumour region is combination of the intensities and the intensities of this tumour region are spreading irregularly. In simple words, the boundary of a tumour is mixing with other normal tissues, where this scenario shows the illusion of a variation of intensities in the region. In fact, this region is the formation of nonuniform intensities. Therefore, a RE is required which improves the visual challenge. The suggested method is effective with RE. The method calculates the confidence score and then converts it to confidence region. Hence, the confidence region is drawn with an effective contour. In more lines, the level set method is applied to the boundaries of the tumour. Hence, the RE determines the tumour region.

If we want to see the context of the problem, it is problematic to extract the brain tumour region from the MRI due to the existence of the varying intensities. The tumour intensities are clearly visible from the image of the brain obtained. Nonetheless, there might exist areas that are ambiguous due to the varying tumour intensities that are spreading out; thus, they cannot be visualised. Hence, it is essential to identify these ambiguous varying tumour intensities. The main objective of the work is to develop the region of interest of brain tumour MRI image method by using confidence score for identifying the variation of intensities. Open-ended questions are how to perform detailed variation of intensities in enhancing the tumour core region? Confidence region contour detection (CRCD) includes detail of contour algorithm to receive an intensity-adjusted image and then identifies the blob and the calculated area of the resultant biggest blob. It also highlights the close contour with confidence region around the tumour boundary and level set methods in image extract edges, and at last confidence region is separating tumour boundaries in tumour growing region.

## 2. Related Work

Region extraction plays an important role for preprocessing of the segmentation. RE is elaborate which is before step of segmentation. In the referenced article, a one-step ahead of enhancement is proposed where spackle entropy noise is handled as compared to the OTSU algorithm [1]. In this referenced article, a Sobel improved edged detection algorithm is proposed which draws close contour to extract the accurate region of a brain tumour [2]. In thisstudy, firstly, they preprocesses the image and handle the noisy images with localisation; secondly, they extract the features and finaly performed the classification [3]. In another referenced study, different modalities have been visually analysed and it has been found MRI segmentation is more challenging for methods due to low pass and high resolution [4]. In another published study, region of interest is important, and tumour shape is enhanced with multi-parameters like the classification of an image pixel to estimate the region of interest [5]. In this referenced proposed algorithm, the bias correction field was discussed which estimates the nonhomogeneous intensities using two multiplicative components, and the technique includes a biased correction field [6]. Recent referenced research suggests a technique for biomedical applications, and the application is made to check the active contour internal energy as well as external energy using the Marr method, gradient vector flow, and vector field convolution methods [7]. In this referenced study, a multi-level threshold OTSU enhances the image with determining significant intensity point and assigns them threshold, after combination results of algorithm are enhanced [8]. In another referenced study, image enhancement is performed with denoising and water-shedding, visual analysis of the image is performed by connected component, and images are also evaluated [9]. In another referenced study, a histogram is used which is a formal analysis of the image, and the intensity of the brain tumour image is corrected by applying standard deviation. This step is normalising the image intensities checked. The white portion of the tumour region is extracted and easily visible [10].

In this referenced study, MICCAI BraTs brain tumour dataset images which are already processed and the skull of the image are removed by the ITK tool kit [11]. In another referenced study, region scale fitting is improved where edge stop function extracts boundaries of noisy image, and after the RSF technique, the noise of the region is removed [12]. In another referenced study, FCM along with feature is used to enhance the edges of the brain tumour image [13]. In this referenced study, the method smoothly detects the inner contour versus outer contour by checking the start and end of the contour point. The background is evolving from region of interest (tumour region) smoothly like background of image removed where foreground of image becomes more highlighted [14]. In another referenced study, edges of brain tumour are more improved; therefore, region of tumour becomes more highlighted [15]. In this referenced study, edges of region of interest (ROI) are detected through the Bayesian contour, and it also gives measurement of tumour area and volume of ROI [16]. In this referenced study, hyperintense lesion is dealt with combination of Gaussian mixture model with synthetic image and support vector machine [17].

In this referenced study, more focus has been made on multi-contrast scan in which variation of tumour stages is presented, 65 algorithms have been tested, and not even one is good for identification of variation; in this referenced study, accurate identification of brain tumour is performed through combination of region growing segmentation, grey-level co-occurrence matrix GLCM feature extraction, and classification through probabilistic neural network (PNN) [18, 19]. In this study,the authors stated that the algorithms produced good results to classify the intensties, but still the problem of region identification unsolved [20]. In this referenced study, enhanced Darwinian particle swarm optimisation is compared with particle swarm optimisation (PSO) for enhancement of brain tumour image; these techniques are identifying the region [21]. In this referenced study, nonuniform intensities are standardising with setting of landmarks and also form a piecewise graph where nonuniform intensities are handled [22]. In this referenced research, glioma feature detection is classified by using Gaussian mixture model. The detection is classifying the tumour region for classification [23]. In this published study, hybrid of two classifiers as multi-layer perceptron and C4.5 decision tree algorithm gives the best classification of tumorous slices and also for nontumorous slices [13]. In this referenced study, initial learning rate improves the performance of SOM for dataset classification and better results of region identification are produced [24]. In this referenced study, the combination of feature is identified for ROI [25]. In this referenced study, segmentation is achieved with combination of unsupervised learning and features [26]. In this referenced study, better tumour detection is found with flavour of SVM classifier for Harvard brain tumour image [27]. In another referenced study, SOM pixel labelling with reduced cluster and deterministic feature clustering give better segmentation [28–31]. Medical imaging is an important field for identification of disease, so doctor can take better decision in perspective of intelligent computation [32–37]. Hence, above state the of art has not enlightened the RE of brain tumour identification, but identification of tumour region is still a challenge for variation of intensity region. The scope of the mentioned issues is crucial, as the issues are opening new horizon which need inventions and improvements. The MRI image region extraction was executed through confidence interval, but it was inadequate for the identification of complete tumour pixels. These pixels are required in enhancing tumour core. If tumour pixels can be identified, the probability of tumour region identification increases. Therefore, in this study by [1], the confidence region was needed for the accurate identification of enhancing tumour.

## 3. Material and Method

### 3.1. Material

**Figure 1:**
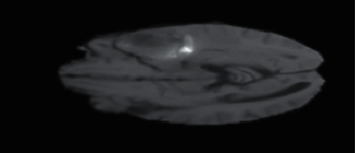
Image of flair.

**Figure 2:**
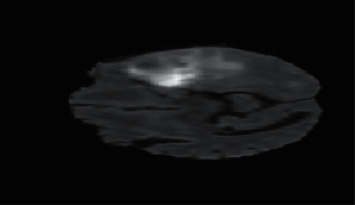
Image of T1.

**Figure 3:**
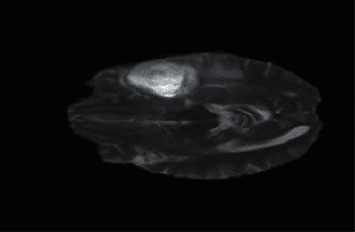
Image of T2.

**Figure 4:**
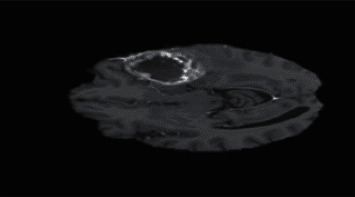
Image of T1Ce.

Tumour identification is possible through confidence region contour detection. Different images are analysing from MICCAI BraTs brain tumour dataset.  Figure 1 shows image of flair, Figure 2 shows image of T1, Figure 3 shows T2, and Figure 4 shows T1Ce. Figure 2 (T1 image) pertains intensity issue. The main purpose of the study is to identify expected tumour region with accuracy. From dataset, each patient has 155 MRI images. Each image has combination of intensities.

From image number 65 to 74, tumour is completely visible. For each patient, ten different tumorous intensity regions are determined from ten abovementioned text. After visual analysis, it is observed that every image has different patterns of intensities. From the pattern of intensities, connected components are determined. Biggest tumour-connected component is selected from individual image. In each image, biggest connected component is called as biggest blob. Tumour biggest blob represents intensity region or tumour region. This process is repeated for rest of images. At end, when these ten images are combined, it is observed that tumour region is growing. Tumorous region is derived from different intensities. This region is called resultant biggest region. Tumour is identified from resultant biggest blob but needs to extract the boundaries of the tumour. Contour is drawing the boundaries of resultant biggest blob. Resultant biggest blob is lineated through the level set method. Using level set, tumour boundaries are extracting accurately. Accurately extracted boundary image is further sent to confidence region. Confidence region is the process of accurate tumour region identification.

### 3.2. Method

The proposed method is named as confidence region contour detection (CRCD), and its detail is mentioned in subsection 3.1.

#### 3.2.1. Confidence Region Contour Detection (CRCD)

In this section, a confidence region contour detection method is discussed in detail. This novelty method gives the exact combination of the biggest blob (connected components). From Figure 2, problem image shows the enhancing tumour growing region. The suggested method is extracting tumour region with the following steps, and it determines the biggest blob area. This blob region is the combination of the maximum connected component area. Connected component areas are consisting of maximum tumour pixels. Figure 2 shows the variation of tumorous intensities.

The values of resultant biggest blob produce the confidence score. This score is transforming to confidence region. The real purpose of transformation for confidence region from confidence score is to get more meaningful information related to tissue. This soft computing of brain image is examining important features. The difference can be compared from the input image information. In fact, this process is a pattern recognition process. Proposed method minimises the error rate of pixel classification, and this classification occurs among the group classes. With combination of thresholding and statistical processing along analytical decision-making, the confidence region classifies pixel more accurately.

(1) $$\begin{array}{c} A1\;=\text{ā}-1.96*\sigma \sqrt{ia}1ja1, \end{array}$$

(2) $$\begin{array}{c} A1<\mu , \end{array}$$

(3) $$\begin{array}{c} A2=\text{ā}+1.96\sigma \sqrt{ia1}ja2, \end{array}$$

(4) $$\begin{array}{c} \mu <A2. \end{array}$$

Equation (1) represents one class of pixels. Equation (3) represents mean values of pixels, and *μ* represents intermediate value. Equation (1) and Eq. (4) can be seen as comparison among A1 and A2.

**Figure 5:**
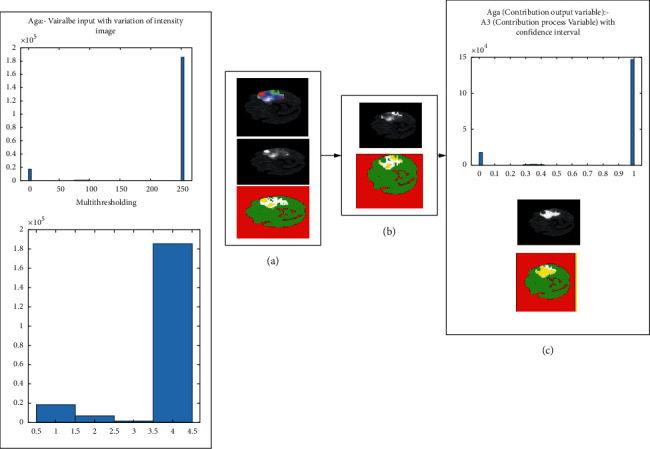
Confidence region formed after passing through the procedure of conversion from confidence score.

The reason to mention Figure 5 here is to show the mind map of confidence region; this mind map figure helps in simplifying the explanation of forthcoming steps. Referring to Figure 5(a), the confidence score is obtained from the biggest blob values. Using the aforementioned equation, the confidence score was transformed to the confidence region. Figure 5(a) shows the histogram of confidence score; it represents the distribution of intensities. From the confidence score, the confidence region is extracted. Figure 5(b) represents another histogram; it shows optimised intensity image distribution, and this histogram shows the confidence region intensities. Figure 5(c) shows extracted confidence region.

**Figure 6:**
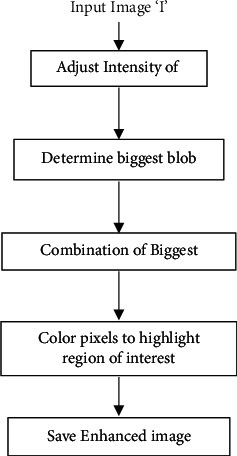
Workflow of confidence region with contour.

The process from confidence score to confidence region consists of standard deviation of the entire image population with relationship to each pixel. The input image is divided into *n* sub-images. Each sub-image Cartesian coordinate of the pixel is related to the greyscale pixels. From equation (1), the confidence interval evaluates the pixel in the sub-image. Then, all the sub-image domains are combined. In Figure 5, the histogram was produced with the confidence region. After the confidence region transformation, the multi-level threshold formed the classification of pixels based on their region. The histogram of confidence region was optimised due to confidence mechanism and tumour RE.  Figure 6 shows the process of confidence region of the resultant biggest blob.

*α* represents brighter intensity tissues of a brain tumour in equation (5). For *α*, certain intensity is determined with visual analysis and *β* determines nontumorous intensities. X is multiplied by *α*, which means that *x* holds the multiple variations of tumorous tissues for *α* (tumorous cells). *β* is added to *α*x, and it represents nontumorous tissues along normal tissues. Therefore, intensities of normal tissues as well as nontumorous tissues are adjusted in the image. Nonenhancing tumour core and tumour homogeneous intensities are needed to separate to a certain level. *α* gives the contrast adjustment, and its value lies between 0 and 1. In particular, the confidence region (CR) enhances the information, *α*x is a parameter, and this parameter is a combination which gives blob area. In brief, this area consists of patterns of connected intensities. Altogether, these patterns are enhanced with maximum pixel value, and these values are more brightly coloured. The confidence region is achieved in the following steps.

*(1) Biggest Blob Area of an Input Image*. Tumour identification issue remains in the adjusted intensity image. As a result, tumour intensities at tumour boundaries mix with normal tissue intensities. Meanwhile, several steps require separating the tumour from other tissues. At first, connected component areas give tumour intensities in T1 sequence. The connected components determine a certain threshold. Thereafter, the algorithm scans the region on the base of the threshold and labels are assigned to each connected region in the area. Later, scanning is performed on the eight connected neighbour pixels in the current area; then, the algorithm assigns labels to the identified areas. Earlier, connected pixels in the area are labelled with a number. Furthermore, in each area, labelled numbers of pixels in the regions are defined with weight and each labelled area consists of pixels with certain intensities. Weight is assigned to every determined area. The biggest area of a connected component in image is assigned with highest priority so that biggest connected component can be easily selected. Therefore, the biggest blob is identified which highlights the area with certain intensity within a greater number of pixels.

*(2) Combination of Biggest Blobs*. The confidence region needs to be determined for identification of tumour region from Figure 2 named as enhancing tumour. Figure 2 shows T1 sequence. Volume of image belongs to sequence of T1 image. Thus, this study defines the biggest blob in the above section. In particular, the biggest blob determines the tumour region, this region is with the maximum area of connected components, and the enhancing tumour intensities are dealt. Accordingly, the maximum region of the connected components is in a specific pattern. Similar patterns of intensities are determined. For instance, image numbers from 65 to 69 (same patient) have similar pattern, whereas 70 to 74 have likewise patterns. Then, all the patterns of intensities are summed up and the resultant is a combination of biggest blobs. Therefore, the combination of the biggest blobs is also known as a resultant blob in this study.

The confidence region with contour detection (CRCD) algorithm is a proposed algorithm, and it identifies the extending tumour intensities. Intensities can separate from normal tissues. The suggested below algorithm tackles mentioned issue, whereas benchmark studies are not dealing the above issue.

The abovementioned detail can be seen in the form of Algorithm 1 and Algorithm 2. Algorithm 1 is giving confidence region of tumour, whereas Algorithm 2 is giving region scale fitting or detecting the contour around tumour area. At first, the image T1 is read from the dataset named as MICCAI BraTs brain tumour dataset. The input image is processed with procedure of intensity adjustment. The limit of intensity adjustment values lies among 0 to 1. Different T1 sequence images of MICCAI BraTs are checked, and it has been known that certain image adjustments of limit of intensity of tumour cells are existing from value of 0.36 up to 0.78. The brightness of the tumour improves, but it is still important to depict the growing region. Intensity adjustment is improving the connected components. Thereafter, all the connected components are scanned throughout the image. The connected component term is also known as a blob. In above detail, we have seen the process of calculation of biggest blob. The process of resultant biggest blob is also discussed in above. Then, a certain RGB colour is assigned to this biggest blob. Consequently, this biggest blob is added to a defined array. Afterwards, the next tumour image belongs to the same patient it selected from the dataset. This image also belongs to same patient but has different certain patterns of intensities. The same procedure repeats, while, nevertheless, the last image is processed. The procedure repeats with all images (belonging to the same patient) with a specific pattern of intensities. Every biggest blob pixel is added to a defined array. In the end, one array is finally obtained which consists of a different pattern of intensities. In fact, this is a resultant image of the biggest blob from various patterns of intensities of the image. Specifically, this resultant biggest blob image is a confidence score image with the maximum pixel of a tumour. This resultant image of the biggest blob sends to the second portion of the algorithm named as a level set algorithm; a set of parameters form a contour. Earlier, this part of the algorithm defines the contour around the tumour position. Therefore, the algorithm sets scaling from a minimum to a maximum row and in the same way a scale from a minimum to a maximum column around the resultant biggest blob. Hence, this subsequent procedure captures the resultant biggest blob and draws a contour around the tumour. Naturally, the level set algorithm convolves the tumour with the Gaussian low pass 13 ∗ 13 kernel. Thereafter, the region scale fitting (RSF) algorithm is used to localise the inside as well as the outside of the tumour boundaries. Data force parameter of RSF detects the edges and boundaries of the tumour. Novel Equation (1) to Equation (4) are premap of new region extraction method, and more motivation is there from Algorithm 1 and Algorithm 2. Algorithm 1 and Algorithm 2 are in more explainable fashion in this study by mean of abovementioned equations if we see the one proposed study [29].

In this last portion, output image (confidence score) is transformed to confidence region using confidence interval. In the first step of transformation, standard deviation of image is calculated. In the second step, confidence interval is defined, and this interval classifies the pixels. Image is divided into four parts. In every part, mean value is calculated. Confidence interval checks every pixel value. If pixel value is greater than mean value, then original pixel value is assigned; in else case, mean value of pixel is added to original pixel value. In another case, pixel value is less than mean value and pixels are assigned with mean values. Finally, all tumour edges are extracted by the confidence region contour detection algorithm. In this paragraph, the algorithm is discussed at a low level. The algorithm works step by step. DWT is applied for identification of confidence region. Lastly, multi-threshold process classifies the pixel. The algorithm's main portion which is confidence region with contour detection takes an image from the dataset and finds out confidence region with step-by-step procedure. Maximum tumorous pixels give an account of the confidence region, and this study identifies the tumour intensities in confidence regions. Hence, all maximum expected tumour intensities are determined from biggest blob.

*(3) Colour Pixels in Region of Interest, Final Biggest Blob, and Comparison to Ground Truth Reality with Confidence*. From the above section, this study finds the combination of the resultant blob. Moreover, it is important to check each individual biggest blob area and then combined to resultant image, and this process is repeatedly combined until resultant biggest blob area image is not former. Specific colour of RGB is assigned to each blob. Blob regarding discussion is done before. Different colours of RGB are assigned to every pattern of intensity. It can be also observed that each pattern has different specific RGB colours in the combined biggest blob. The RGB colour shows the significance of blobs. RGB-combined biggest blob region is called the confidence score. The overlap of this confidence score can be compared with the ground truth reality image. This overlap of confidence score image and ground truth image shows result with accurate tumour shape and region of enhancing tumour. In conclusion, the enhancing tumour region intensities are identified with a confidence score.

## 4. Confidence Region and Contour Detection Algorithm

**Figure 7:**
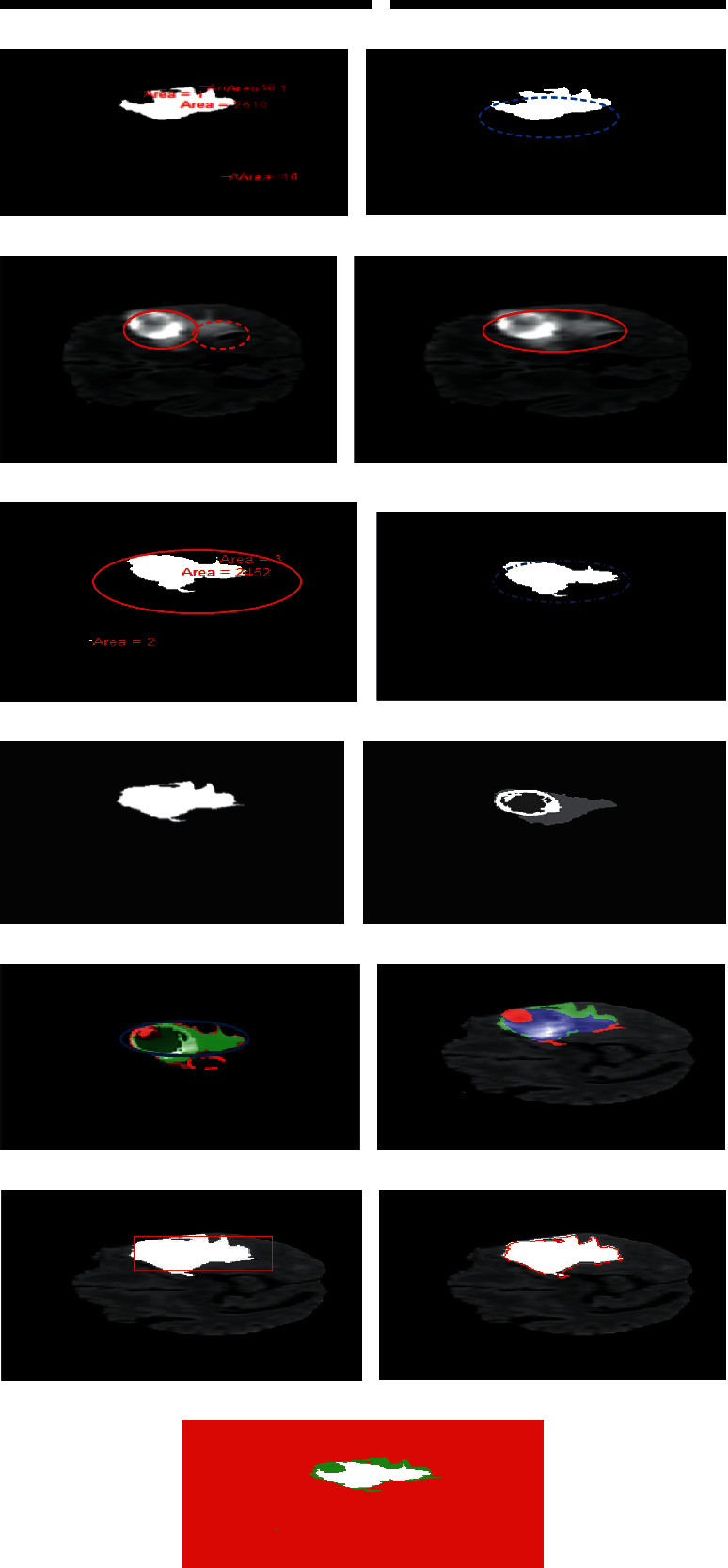
Confidence region contour detection. (a) T1 sequence original image with a pattern of intensity, (b) intensity adjustment of one pattern of intensity, (c) blob areas from certain intensities, (d) selected biggest blob, (e) intensity adjustment of an image from a particular pattern of intensities; they are from 68 towards 74, (f) intensity adjustment of 69 towards 74, (g) blob area in slices from 70 to 74, (h) biggest blob from Figure 12, (i) combination of biggest blobs from slice 65 to 74, (j) ground truth reality image of slice 74 17, (k) overlap portion of (i) and (j), (l) the confidence region of tumour highlight with other cells, (m) contour drawn around tumour boundaries, (n) region scale fitting used for the extraction of tumour, (o) confidence region contour detection.

Below algorithm is named as the confidence region with contour detection (CRCD). This algorithm is explained in different paragraphs. The purpose of the algorithm is to identify the tumour boundary in image. When tumour intensity is extending, it can be seen in Figure 7 a and *b* part. Algorithm identifies the resultant biggest blob. Another part of the algorithm on the next page is named as the level set algorithm with setting parameter over contour. And then lineate the tumour boundaries using the level set algorithm.

## 5. Results and Discussion

The results of contour enhancement are obtained with a parameter known as resultant biggest blob (RBB). The RBB counts the score, and a contour is drawn around the boundaries of the RBB. The level set method is used to fit the scale of tumour. A contour is drawn from the maximum column to the minimum, and then from the minimum row to the maximum. The level set region scalable fitting is used for delineating the tumour boundaries. The number of tumour-detected pixels gives a confidence score value; this value is further transformed to Confidence Region. The Tumour Confidence Region is compared with ground truth reality images. The ground truth reality images are available in the dataset. Four testing parameters have been used to compare the RE results with the ground truth reality image

Figure 7(a) shows an input image of the original flair sequence with certain patterns of intensities. In particular, the dotted red line represents a enhancing tumour core, whereas the red line circle shows the tumour contrast region. Moreover, normal cells are linked with weak boundaries. Hence, Figure 7(a) shows a problem image with enhancing tumour core.  Figure 7(b) shows the intensity adjustment of an input image in Figure 7(a). The result of the intensity adjustment is a tumour region which becomes more contrasted, when it is compared to the enhancing tumour core region. It has been the tumour cell mixes with the other cells. It is important to enhance the intensities of the tumour. From Figure 7(c) to Figure (d), tumour region is identified step by step.  Figure 7(b) shows the step where the tumour areas rely on the connected components. These connected components are called blobs, and areas of blobs are calculated by assigning those labels. Moreover, the red line in Figure 7(c) represents the biggest blob area. Adjacent to the biggest blob, there are the other blob areas with measurement of 16 mm, 10 mm, 2610 mm, etc. As mentioned earlier, enhancing tumour core MRI images have a pattern of intensity. Furthermore, Figure 7(c) shows more different regions of the blob. Next, a higher weight is assigned to the biggest blobs as the study shows in Figure 7(c). In above described context, Figure 7(d) gives an illusion of the biggest blob under the blue dotted line. This dotted blue line is a result of weighted biggest blob area. A pattern of the intensities from image number 65 to 69 shows similarity with very small intensity variations. Hence, the biggest blob is selected from them and they are considered as one pattern of the intensity image. This pattern has identified region of interest from one pattern. In Figure 7(e), another pattern of intensities is determined. This case is like Figure 7(a) but with different intensities. Similarly, the above image has the issue of enhancing tumour core like Figure 7(d). This figure shows enhancing intensities. In the above image, dotted lines show enhancing core whereas the plane red line shows the intensities of the brain tumour.  Figure 7(f) shows the intensity adjustment. Specifically, this step aims to increase the contrast of the brain tumour so that the tumour portion can be separated from the enhancing tumour core (enhancing tumour intensity) as compared to normal tissues. The deep red line shows the intensity adjustment region. In brief, Figure 7(f) shows the same presence of abovementioned issue. Like as the above discussion, patterns of intensities are the same with small variation. Therefore, the intensities are likewise from image 69 to 74 with little variation. Therefore, this pattern of intensities still needs identification. In Figure 7(g), the same steps of Figure 7(c) are repeated but this resultant image keeps a different pattern of intensities. A connected component (blobs) determines certain intensities on the base of a certain threshold. In this way, different regions (areas) of the blob are determined. In above image, one area is 2 mm, the second one is 3 mm, and the third one is 2152 mm. The deep circle red line is drawn around the biggest blob area.

 You can see Figure 7(f) represents another different pattern of intensities. After experimentation and comparison, the largest region (area) is selected as shown in Figure 7(g). The figure shows the identified shape of the tumour. The reason for selecting the largest area is to identify tumour region issue when region is growing intensity. The issue is resolving on one stage. From experimentation, it is observed that Figure 7(d) determines the biggest blog (shape of tumour) from one pattern of intensity. Similarly, Figure 7(h) produces the biggest blob (shape of tumour) from other patterns of intensities. Later, they are combined as shown in Figure 7(i) and the result is a confidence region score. This is also called the resultant biggest blob area. This area consists of a maximum pixel of tumour shapes. The confidence score determines the image from all patterns of intensities and produces one combined pattern of intensity. Hence, this step solves the issue of enhancing tumour core intensity. In Figure 7(i), the area shows hybrid pattern of intensities.  Figure 7(j) shows the ground truth reality image. In the dataset, every image has a specific segmented image. This image is related to the input pattern of the intensity image. The above under red line segmented tissue image is provided by the dataset. For testing purposes, the provided dataset image is useful in the proposed study.  Figure 7(k) shows the overlap portion between Figure 7(i) and Figure 7(j). Particularly, Figure 7(i) shows a confidence score image which extracts the tumour region, whereas Figure 7(j) shows the tagged or ground truth image of the same patient as well as the same input image. The confidence score image and the ground truth image both are overlapped. In Figure 7(k), the deep blue colour shows the overlap whereas the dotted red line shows the un-overlapped portion. Here, the confidence score is verified.  Figure 7(l) shows that the confidence score determines the tumour tissues along other normal cells. Moreover, the grey matter (GM) cells are normal brain cells whereas the tumour cells are highlighted with RGB colour. This study shows a pattern of intensities. In Figure 7(k), a different pattern of intensities is identified; therefore, tumour enhancing intensity issue is resolved, and it can be seen in Figure 7(j).  Figure 7(m) shows a contour which is drawn around the resultant biggest blob (tumour), and the tumour boundaries are with white colour and 13 ∗ 13 Gaussian kernels which convolve around the resultant biggest blob. Specifically, the tumour is extracted with proposed confidence region (CR). The contour predicts high contrast intensities within a scale of the minimum column to the maximum column as well as the minimum row to the maximum row. Hence, contour detection is performed around the confidence score (CS) resulting in the biggest blob. This technique focused on the region of tumour boundaries and edges. In Figure 7(n), the level set algorithm named as the region scale fitting (RSF) is used for the lineate tumour portion. This level algorithm relies on contour detection (CD) of confidence score (CS). Using this algorithm, a red line is drawn over the tumour boundaries. These extracted boundaries and edges can be compared with the input image. Within hundreds of iterations, inner intensities and outer intensities are separated by the level set algorithm; then, a line is drawn among them. This determines, from Figure 7, the tumour boundaries that are separating the regions step by step. In Figure 7(o), it shows the transformation of confidence score to confidence region. This step is the main contribution step.  Figure 7(m) shows prestep of confidence region. A confidence interval has been defined. Defined interval has classified the pixel. The process of confidence region transformation using confidence interval can be seen from Equation (1) to Equation (3), and detail can be also seen in Figure 5. The result of confidence interval determines the confidence region. Dice over index (DOI) is more than 95 percent, and Jaccard index is also more than 95 percent.

From Table 1, column (a) is a list of input images from volume 1 to volume 10 from sequence T2, column (b) consists of CRCD proposed technique and is applied on input image, column (c) is a list of images consisting of ground truth images. In Table 2, average values of testing parameters are calculated for sequences of dataset named as flair, T1, T2, and T1CE where DOI is 0.97, JI is 0.94, MSE is 1.24 db, and PSNR is 17.45 db. Average results are mentioned in Table 2; results in Table 2 are calculated for extracted region which can be seen in Table 2, these results indicate confidence region for T2 where (a) is input image whereas (b) is RE images after execution of steps for confidence region and contour detection process. Confidence region is the contribution, and results can be seen from Table 2.

## 6. RE Capitulation

**Figure 8:**
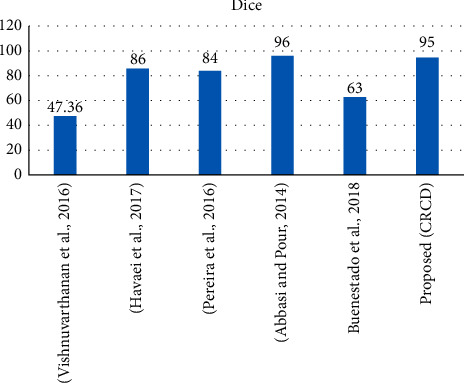
Overall comparison of Jacquard values with an approximation.

**Figure 9:**
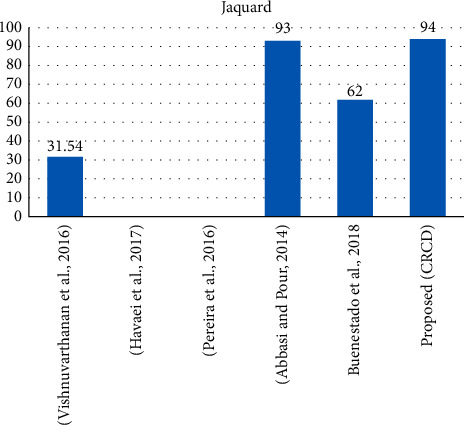
Overall comparison of Jacquard values with an approximation.

For RE, a different technique has been proposed using hybrid unsupervised learning like SOM-KMEAN and SOMFCM, whereas performance statistical evaluation is best one, and confidence interval [1] was proposed but still the RE results are not good like 63% for DOI and 62% for JI. The results of this study are 95% for DOI and 94% JI. It can be seen in Figures 8 and 9.

## 7. Conclusion

The whole work of RE is concluding here with solution of extending tumour intensity and is identifying the tumour in image using the resultant biggest blob. Proposed method contour detection with confidence region (CRCD) highlights the boundaries of the tumour tissues from the complex case of extending tumour core. The resultant biggest blob is a output parameter of algorithm, and it results with identification of tumour with maximum region with the CR. Region scale fitting (RSU) is excelling lineation of tumour boundaries. Hence, the proposed method CRCD is extracting the tumour region. Average value is as follows: DOI is 0.97, JI is 0.94, MSE is 1.24 dB, and peak signal-to-noise ratio is 17.45 dB. Consequently, the objective is an RE that is achieved. This work is more concerned on region extraction (RE); RE solves the issue of variation of intensities, whereas intensities spread towards boundaries known to be extreme intensities; then, there is room still of improvement of mentioned case. This issue will be solved with concept of segmentation.

## Figures and Tables

**Algorithm 1:**
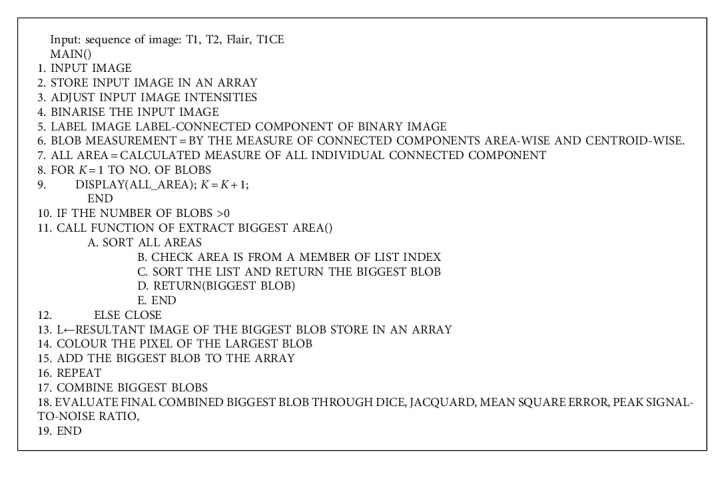
Contour detection with confidence region.

**Algorithm 2:**
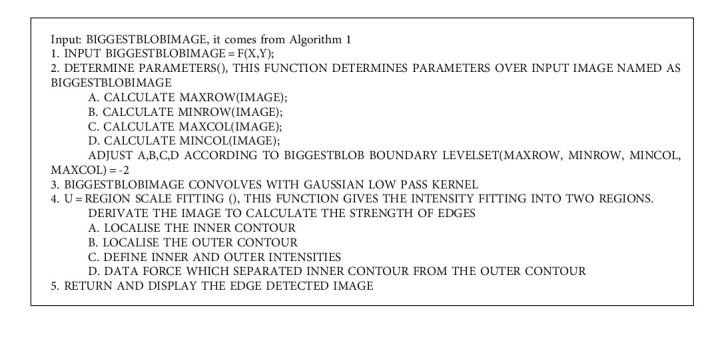
Level set algorithm with setting parameter over contour.

**Table 1:** Results of CRCD for T2 sequence.

Name of image	Input image (a)	CRCD image (b)	Ground truth image
Vol_1_T2	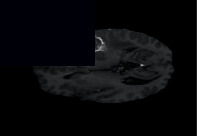	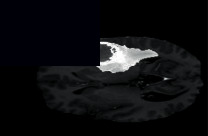	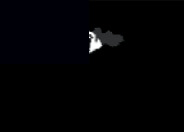
Vol_2_T2	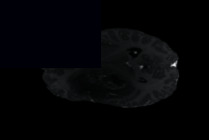	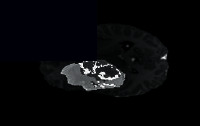	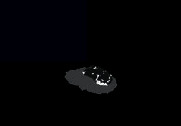
Vol_3_T2	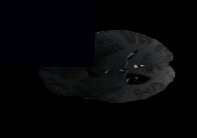	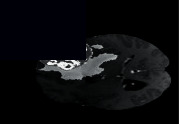	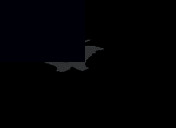
Vol_4_T2	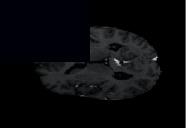	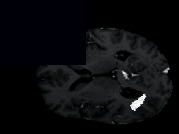	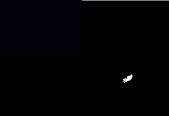
Vol_5_T2	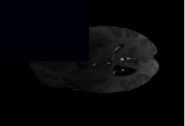	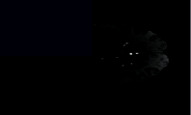	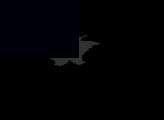
Vol_6_T2	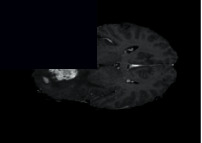	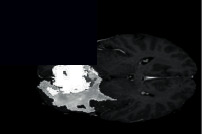	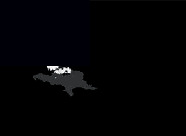
Vol_7_T2	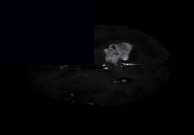	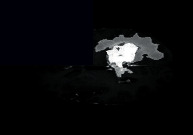	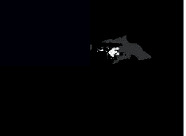
Vol_8_T2	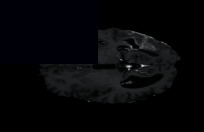	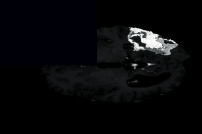	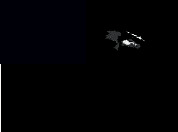

**Table 2:** Results of CRCD.

Dataset	DOI	JI	MSE	PSNR
Vol_1_flair	0.98	0.95	0.90	18.76
Vol_1_T1CE	0.97	0.95	0.86	19.05
Vol_1_T2	0.97	0.94	0.75	19.05
Vol_1_T1	0.98	0.95	0.91	19.05
Vol_2_flair	0.96	0.94	17.57	0.91
Vol_2_T1CE	0.97	0.95	0.72	17.57
Vol_2_T2	0.97	0.93	0.81	17.57
Vol_2_T1	0.97	0.94	0.84	17.57
Vol_3_flair	0.98	0.93	0.93	18.58
Vol_3_T1CE	0.96	0.93	0.76	17.24
Vol_3_T2	0.96	0.91	0.76	17.24
Vol_3_T1	0.96	0.92	0.83	17.24
Vol_4_flair	1.00	0.99	0.92	18.66
Vol_4_T1CE	1.00	0.99	0.90	18.66
Vol_4_T2	1.00	0.99	0.79	18.66
Vol_4_T1	1.00	0.99	0.91	18.66
Vol_5_flair	0.96	0.93	0.84	17.24
Vol_5_T1CE	0.96	0.93	0.76	17.24
Vol_5_T2	0.96	0.93	0.76	17.24
Vol_5_T1	0.96	0.92	0.83	17.24
Vol_6_flair	0.96	0.92	0.79	16.06
Vol_6_T1CE	0.95	0.91	0.70	16.06
Vol_6_T2	0.95	0.91	0.76	16.06
Vol_6_T1	0.96	0.92	0.87	16.06
Vol_7_flair	0.97	0.94	0.83	18.89
Vol_7_T1CE	0.96	0.93	0.74	18.89
Vol_7_T2	0.96	0.92	0.87	16.06
Vol_7_T1	0.96	0.93	0.92	18.89
Vol_8_flair	0.98	0.97	0.86	16.17
Vol_8_T1CE	0.98	0.96	0.74	16.17
Vol_8_T2	0.96	0.93	0.73	18.89
Vol_8_T1	0.98	0.97	0.92	16.17
Vol_9_flair	0.98	0.95	0.89	19.18
Vol_9_T1CE	0.97	0.94	0.84	19.18
Vol_9_T2	0.97	0.94	0.72	19.18
Vol_9_T1	0.98	0.95	0.70	19.18
Vol_10_flair	0.98	0.96	0.84	18.32
Vol_10_T1CE	0.98	0.96	0.88	18.32
Vol_10_T2	0.98	0.96	0.77	18.32
Vol_10_T1	0.98	0.96	0.95	18.32
Average	0.97	0.94	1.24	17.45

## Data Availability

Data used in this research are available upon request from the corresponding author.
